# Introducing an attitude-based approach to emotional intelligence

**DOI:** 10.3389/fpsyg.2022.1006411

**Published:** 2023-01-16

**Authors:** Jo Maddocks

**Affiliations:** ^1^Department of Neuroscience, Psychology and Behaviour, University of Leicester, Leicester, United Kingdom; ^2^Talogy, Guildford, United Kingdom

**Keywords:** attitude, emotional intelligence, ethics, dual-processing, emotional efficacy, self-development, non-linear measurement, unconditional positive regard

## Abstract

Emotional intelligence (EI) was originally conceived as an ability, followed soon after by mixed, competency and trait theoretical models, broadly described as emotional efficacies (EE). Several models have attempted to integrate both approaches, with different views on whether EI and EE operate in sequence or parallel. One reason for this may be that EE constructs are given the same ontological status whether they represent underlying attitudes, such as self-regard, or behavioral competencies, such as assertiveness. In this paper, it is proposed that attitudes may predominantly act as underlying antecedents of ability-EI and behavioral-EE. Five benefits of this approach are drawn out that help to address some key concerns with current models and measures of EI and EE. First, the inclusion of implicit and explicit attitudes within integrated models of EI/EE would support the dual-processing of conscious and automated processes. From this, an attitude-based dual-processing framework for EI/EE is recommended. Second, the concept of Unconditional Positive Regard (UPR) for self and others, is identified as a potential attitude that may underpin the two core pillars of intrapersonal and interpersonal EI/EE. Third, UPR attitudes would provide an ethical basis for EI/EE that may support ethical and prosocial behavior. Fourth, UPR attitudes may differentiate between the optimal and sub-optimal elements of EI/EE. Fifth, an attitude-based approach to EI/EE may be more aligned with EI/EE being developmental than are the more static ability or trait-based models of EI/EE.

## 1. Introduction

Over the last 30 years, research into emotional intelligence (EI) has taken two distinct and contrary pathways dominated by a few prominent models. One examines EI as an ability (Mayer and Salovey, 1993), measured as maximum performance. The other describes EI as a mixed array of affect-related traits (Furnham and Petrides, 2003) or competencies (Boyatzis and Sala, 2004), measured as typical performance. In recent years, there has been a wider recognition from theorists of both approaches that the term “emotional intelligence” (EI) may be reserved for the collection of cognitive and emotional processes aligned with the ability model, while emotion-related behaviors and traits are labeled something other than “intelligence” (Mayer et al., 2008) such as “emotional and social competencies” (Boyatzis, 2007), “affect-related personality” (Hughes and Evans, 2018. p. 5) and “trait emotional self-efficacy” (Petrides et al., 2016, p. 339). From here on in I employ the term “emotional efficacies” (EE), a label seen as acceptable across different sides of the ongoing debate that surround EI (Dasborough et al., 2021, p. 11).

This raises the question of how these two taxonomies of EI and EE are related. In a review of three decades of EI research, Keefer (2015) concludes that the ability and trait approaches to EI have become recognized as complementary rather than competing or contradictory, reflecting a shift toward more integrative approaches. These approaches take different forms. Some present EE traits and competencies as outcomes of EI ability (Mikolajczak, 2009; Cherniss, 2010; Joseph et al., 2015; Boyatzis, 2016; Drigas and Papoutsi, 2018). Others consider EE traits to be antecedent to EI ability (Seal and Andrews-Brown, 2010), while later models propose that some or all components of EE and EI may run in parallel (Hughes and Evans, 2018; Vesely-Maillefer et al., 2018). Despite this move toward integration, other scholars lean toward their separation, noting the low correlation between EI and EE measures (Brackett and Mayer, 2003; Warwick and Nettelbeck, 2004), with a foothold in either ability-EI (Mayer et al., 2008; Dasborough et al., 2021) or the trait-EE camp (Petrides, 2010). The integration of EI and EE models is gradually advancing as a field of theoretical research but is still to establish itself with a consistent and unifying approach. It is not yet agreed whether EE, or components of EE, are antecedent, parallel to, or consequences of ability-EI. This may in part be due to the potential overlap and breadth of constructs found across different models and measures of EE traits and EE competencies. For example, the Trait Emotional Intelligence Questionnaire (TEIQue; Petrides et al., 2007) has many similar scales labels (such as Trait Empathy, Trait Optimism, and Adaptability) to the Emotional and Social Competency Inventory (ESCI; Boyatzis, 2007; such as Empathy, Positive Outlook, and Adaptability). One reason it may be difficult to draw clear distinctions between EE traits and competencies is that they are given the same ontological status. For example, Bar-On (1997), defines EQ (EE) as “a cross-section of interrelated emotional and social competencies, skills and facilitators…” but does not explicitly differentiate between these components. Facets such as Self Regard and Problem Solving, as measured by the Emotional Quotient Inventory (EQI; Bar-On, 1997) are given the same relative importance, with no attributional or causal relationship made between them. Similarly, facets from the TEIQue are collectively defined under broad descriptions such as “a constellation of emotional self-perceptions” (Petrides et al., 2007, p. 26) and “affective aspects of personality” (Petrides et al., 2016. p. 336), with no distinction made between emotional processes such as Emotional Regulation and dispositional traits such as Optimism. This reflects a wider concern about EE models being a “catch-all label” and a “grab bag” (Murphy, 2006; Joseph and Newman, 2010) containing a diverse range of knowledge, skills, abilities, and other characteristics (KSAOs; Joseph et al., 2015).

One way forward would be to differentiate the ontological status of EE facets to distinguish those facets that are more profound and deterministic of EI-related outcomes from those that are specific behavioral manifestations of EI. For instance, the scale facet of Self Regard reflects an individual’s underlying self-concept that may manifest broadly in a person’s emotional, cognitive, and behavioral response to events (Diener and Diener, 1995; Marsh and O’Mara, 2008). In this article, it is proposed that greater attention is given to an individual’s self-concept, in particular, their implicit and explicit attitudes as important determinants of both ability-EI and behavioral-EE.

## 2. Attitudes as a foundation for EI and EE

Attitude is frequently referenced in relation to EI and EE models, although this relationship is mostly described in broad and non-specific terms. In their integrated model of EI, Seal and Andrews-Brown (2010, p. 147) suggest that “having the right attitude predisposes the use of certain skills, and that both attitude and skills are influenced by ability.” Similarly, Vesely-Maillefer et al. (2018) reference KSA (Knowledge, skills, attitude) taxonomy of Bloom (1976), as analogous to their integrated model of EI. Cherniss and Boyatzis (2013) include a wide array of underlying elements to EI such as “motivation,” “unconscious dispositions,” and “values and philosophical foundations” as foundations of the ESCI model, and Keefer (2015, p. 9) describes self-report scales of emotional competencies as an individual’s “EI self-concept.”

There is substantial evidence that attitudes initiate and influence cognitive and emotional processes. Ajzen (2007) defines an attitude as “a disposition to respond favorably or unfavorably to an object, person, institution or event” and most contemporary social psychologists agree that a defining characteristic of attitudes is they are evaluative, i.e., they elicit a cognitive and emotional response (Osgood et al., 1957; Bem, 1970; Fishbein and Ajzen, 1975; Oskamp, 1991; Eagly and Chaiken, 1993). Neuroscientific evidence shows that attitudes, when stimulated, activate the emotional centers of the brain within the limbic system and amygdala (Zald, 2003; Phelps, 2006), giving rise to an evaluative emotional response (Peikoff, 1991), which then exerts influence on thought and behavior (Fazio et al., 1986; Bargh et al., 1992; Baumeister et al., 2007). For example, attitudes influence where we focus our attention (Gwinn and Krajbich, 2020), whether we interpret events through a positive or negative lens (Cacioppo and Berntson, 1994), and how we choose to then respond to the event (Fazio and Petty, 2008). Such evidence would suggest that attitudes may have an important influence on the perception, facilitation, understanding, and management of emotions, as represented in the ability and emotion processing models of EI.

The relationship between attitude and behavior is also well-established (Ajzen and Fishbein, 1977; Fazio et al., 1986; Bargh et al., 1996). A large meta-analytic review found an average correlation of 0.52 between attitude-opinions and behavioral actions (Glasman and Albarracín, 2006) concluding that attitudes influence future behaviors when they are easy to retrieve from memory and stable over time. The notion that individuals seek consistency and balance between attitude and behavior is also central to social and cognitive models of psychology (Festinger, 1957; Heider, 1958; Bandura, 1986; Makin and Cox, 2004), such as self-consistency theory (Korman, 1970) and self-verification theory (Swann, 1992). Lindebaum (2009, p. 233) asserts that “endeavors to stimulate individual’s EI should center upon attitudinal and perceptual changes before behavioral responses can change too.” Cognitive dissonance theory (Festinger, 1957) demonstrates that an individual may feel emotionally compelled to align their behavior with their attitude or alter their attitude to fit with their behavior, to avoid feelings of dissonance and anxiety (Cooper and Fazio, 1984). Wide reaching research into self efficacy (Bandura, 1986) also provides strong conviction that self-belief, a close relative of attitude^1^ greatly influences thought, motivations, and action. The close theoretical and empirical relationship between attitude and behavior is also widely applied within organizational settings to promote job satisfaction, organizational commitment, and personal development (Miao et al., 2017). Given the depth and history of research that aligns attitudes to emotion, cognition, and behavior it is curious that the link between attitude, EI, and EE has not been made more explicit within current models.

On the premise that attitude is intrinsically linked with emotional processing and emotion-driven behavior, it follows that incorporating attitudes within existing integrated models of EI/EE may provide a causal link to EI ability and EE behaviors. This may take the form of attitudes as underpinning antecedents, which influence both ability-EI and behavioral-EE. In this paper, it is proposed that attitudes and an attitude-based approach to EI/EE present several potential advantages that help address concerns with current models of EI/EE. These include:

(1) Greater understanding and separation between the conscious (implicit attitudes) and automated (explicit attitudes) dual-processing of EI/EE.
(2) Foundations to the intrapersonal (internal attitudes) and interpersonal (external attitudes) pillars of EI/EE.
(3) An ethical basis for EI/EE that promotes ethical and prosocial behavior.
(4) Balance between the extremes of too much and too little EI/EE.
(5) A platform that supports the developmental application of EI and EE.

These five benefits will be discussed with recommendations for an attitude-based dual-processing framework of EI/EE that differentiates the potential inputs (attitudes) and outputs (behavioral-EE) of EI (the ability).

## 3. Discussion

### 3.1. A dual-processing model of EI/EE

A concern rarely considered by creators of EI models and measures is the differentiation between conscious and automated processes. Ybarra et al. (2014, p. 96) claim that ability-EI models tend to focus on the conscious cognitive processing of emotions, i.e., awareness, understanding, and management of emotions, but ignore automatic unconscious processes, missing out on an important part of the EI puzzle. For example, the MSCEIT ability instrument (Mayer et al., 1997), has been criticized for tapping into emotion-related knowledge rather than emotion-related ability. As Ashton-James (2003) explains, knowing what one should say, or how one should behave to sustain a relationship in a specific situation (conscious knowledge), does not mean that one will behave this way in practice. Fiori and Vesely-Maillefer (2018, p. 37) describe this as “among the most compelling theoretical challenges we need to address” and argue that incorporating automatic processes into a model of EI is critical because a large portion of social and emotional life is regulated through the deployment of such processes (Bargh and Chartrand, 1999; Kahneman, 2011). Dual-processing models of EI have been advocated by a few researchers (Evans, 2008; Fiori, 2009; Ybarra et al., 2014) and are consistent with wider dual-processing theories of emotion (Baumeister et al., 2007). Fiori (2009) recommends a dual-processing framework of ability-EI that include both crystalized EI (emotion knowledge) and fluid EI (emotional information processing), suggesting that ability-EI measures that focus on crystalized EI may be more suited to predicting “effortful and consciously accessible emotional behavior,” and that measures of fluid EI or emotion information processing may account more for “spontaneous and unintentional behavior” i.e., automaticity. More recently, this has been developed into an integrated model of EI (Vesely-Maillefer et al., 2018) with three components that interact and operate simultaneously (PAT): Processing of emotional information (fluid EI), Ability (crystalized EI), and Traits.

As with ability-EI models and measures, the behavioral-EE approaches make little separation between automatic, habitual behaviors and more deliberate, conscious behavioral actions. The majority of EE trait and competency measures are self-report, which require conscious and accurate self-awareness of one’s behavioral and emotional patterns. However, EE traits and competencies are typically dispositional, habitual, and skill-based concepts, which are likely to be more automated and often unconscious processes.

It is proposed that this important distinction between automatic and conscious processing is incorporated into models of ability-EI and behavioral-EE and that attitudes could form a basis for such an approach. Attitude theory lends itself well to a dual-process approach as a clear distinction is made between *explicit* attitudes, which operate at a conscious level, are deliberately formed, and are easy to self-report, and *implicit* attitudes, which operate at the unconscious level, are involuntarily formed, and are typically unknown to us (Bassili and Brown, 2005). From their review of the literature, Bohner and Dickel (2011, p. 412) comment that “Research on attitudes as precursors of behavior shows that implicit measures of attitude predict spontaneous, less controllable behavior, whereas explicit measures of attitude predict deliberative, more controlled behavior.” One reason why attitudes may have been neglected from EI research is that they are often implicit, and therefore difficult to identify and not easily available through self-report. Implicit attitudes may be seen as templates, patterns (Griffin and Tyrell, 2001), or simulations (Barrett, 2017) against which sensory stimuli are initially matched to invoke emotional, cognitive, and behavioral responses (Fazio et al., 1986; Bargh, 1989; Bargh et al., 1992). For instance, studies have shown that information is sent to pre-conscious regions of the brain (associated with emotion and the limbic system) 0.3 s before reaching higher conscious regions of the brain (associated with cognition; Libet, 1985; LeDoux, 1996). This implies that conscious thinking and the degree of choice a person has over their thoughts, decisions, and actions may be pre-influenced by earlier emotional responses, activated automatically by their implicit attitudes (Bargh, 1989; Bargh and Morsella, 2008). Given that attitudes are intrinsically linked with emotional processing and emotion-driven behavior, and that all three elements (attitude, ability-EI, and behavioral-EE) operate both automatically and consciously, it may be beneficial to incorporate attitudes within a dual-processing framework of EI and EE.

**Figure 1:**
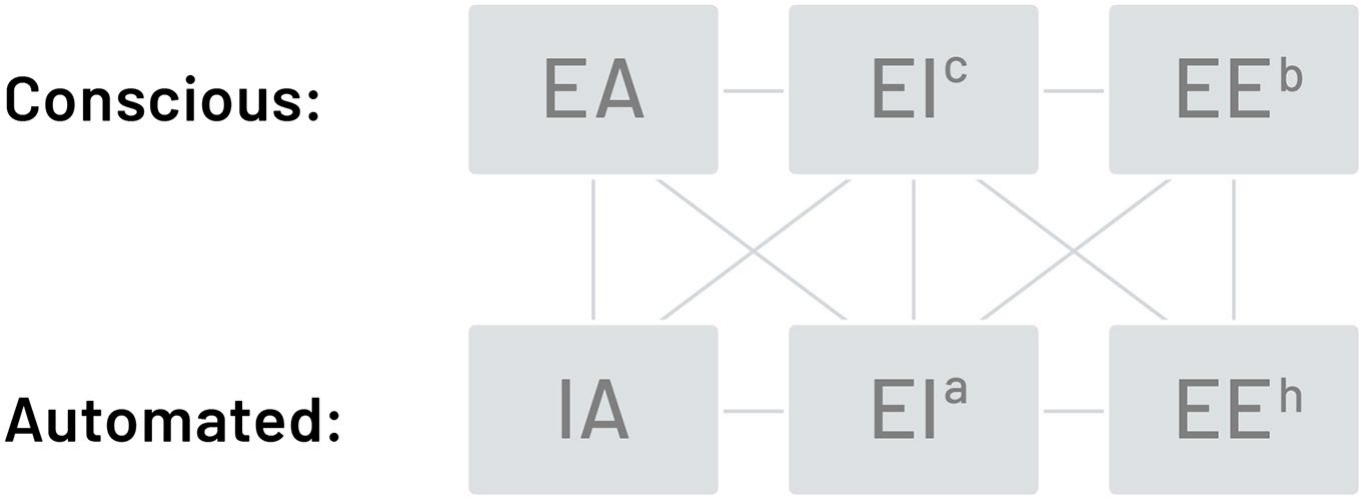
An attitude-based dual-processing framework of EI.

Building upon existing integrated models, this may take the form of two parallel and interconnected streams, conscious and automated, as shown in Figure 1. Each stream would comprise three components: attitudes, ability-EI, and behavioral-EE, with attitudes as antecedent to both ability-EI and behavioral-EE, as indicated by the horizontal lines. In the conscious stream, explicit attitudes (EA) may motivate an individual toward behavioral action and effective management of their emotions (EE^b^). To perform the action they may also draw upon crystallized EI knowledge (EI^c^). For example, an individual may consciously adopt a positive intention (their explicit attitude – EA) to be calm and relaxed, and then embed this attitude by rehearsing an affirmation to themselves, such as “I feel calm, composed, and relaxed” (EE^b^). To further aid relaxation, they may learn a specific breathing technique (EI^c^) which they implement daily (EE^b^).

Through conscious practice, skill acquisition, and habit formation (Fitts and Posner, 1967; Anderson, 1982; Sun et al., 1996), the three components may become proceduralised and automated such that explicit attitudes (EA) gradually become implicit (IA; Gawronski and Bodenhausen, 2006), emotion knowledge (EI^c^) becomes a fluid automated process (EI^a^), and conscious deliberate behaviors (EE^b^) become skillful habits (EE^h^). The process may also be reciprocal such that automatic and unconscious processes become conscious, such as becoming aware of unhelpful behavioral habits (EE^h^) following 360-degree feedback or recognizing unconscious biases (IA) through awareness training. The bi-directional link between the conscious and automated streams is illustrated by the vertical lines in Figure 1. Equally, some elements may be automated and others conscious, as indicated by the diagonal lines, such as an individual who has an unconscious fear of failure (IA) but is acutely aware of feeling anxious while completing tasks (EI^c^) and consequently demonstrates perfectionist tendencies (EE^h^).

As to which stream an individual takes, conscious or automated, may depend on whether the matter is sufficiently familiar and routine to be automated, or challenging enough that it meets the threshold for conscious awareness (Baumeister et al., 2007). Drawing upon the neuroscientific literature, Baumeister et al. (2007, p. 170) contend that “it is mainly the automatic affective responses that directly contributes to causing behavior.” Although the prevailing direction of influence in this model is from left to right (attitudes as antecedent to EI and EE), it is also recognized that there is a reciprocal influence in the opposite direction such that attitudes may shift to be congruent with cognition and behavior (Festinger, 1957; Bem, 1965, 1972). This reciprocal interaction, combined with the interdependence of the conscious and automated streams creates many interconnections between the three components that may form the basis of initial hypotheses for further investigation.

The inclusion of implicit and explicit attitudes as key determinants of EI and EE builds on several integrated and dual-processing models mentioned previously (Ybarra et al., 2014; Joseph et al., 2015; Vesely-Maillefer et al., 2018). It provides an organizing framework for the relationship between conscious and automated processes and differentiates between ability-EI, its inputs (attitude), and outputs (behavioral-EE). It also strengthens the case for differentiating between conscious and automated processing of EI and EE. Including attitudes as antecedents to EI and EE raises the question as to *what* attitudes may facilitate emotionally intelligent processes and behavior.

### 3.2. Foundations for intrapersonal and interpersonal EI/EE

As well as being implicit and explicit, another feature of attitudes that relates closely to EI/EE is the differentiation between self and others. Attitudes may broadly be directed internally, toward oneself and the self-concept, or externally toward a person, place, object, or event (Ajzen, 2007). Similarly, it has become more common within models of EI/EE, to distinguish between the intrapersonal (self) and interpersonal (others) domains (Fiori and Vesely-Maillefer, 2018) with growing evidence to suggest that individual differences may exist between both streams (Mikolajczak et al., 2015; Troth et al., 2018). Ybarra et al. (2014) comment that there is no reason to assume that someone strong in one area of EI, such as being aware of their feelings, will be capable in another area of EI, such as being aware of the feelings of others. Brasseur et al. (2013) postulate that in some cases intrapersonal EI may carry more weight than interpersonal EI (e.g., for managing job stress) but the opposite may be true in other cases (e.g., for building relationships).

An overarching attitude that may underpin the self and other streams of EI/EE is the concept of unconditional positive regard (UPR; Rogers, 1957), which may be applied to oneself (self-regard) or to others (regard for others). Rogers (1959, p. 206) defines Positive Regard as “including such attitudes as warmth, liking, respect, sympathy, and acceptance,” and Lietaer (2001, pp. 92–93) defines Unconditionally as “valuing the deeper core of the person.” For example, caring for someone even when disapproving of their actions, such as a parent’s love for their child, or maintaining feelings and self-worth even when underperforming at work. UPR was later applied within Transactional Analysis theory (Berne, 1964) and defined as “one’s basic beliefs about self and others, which are used to justify decisions and behavior” (Stewart and Jones, 1987, p. 119). Self-regard is conceptually similar to the construct of self-esteem (Blascovich and Tomaka, 1991), which has been intensively studied as a variable of personality psychology (Costa et al., 1991; Judge and Bono, 2001; Zeigler-Hill et al., 2015). A classic definition of self-esteem is “it expresses an attitude of approval and indicates the extent to which an individual believes himself to be capable, significant, successful and worthy” (Coopersmith, 1967, pp. 4–5). Other-esteem (regard for others) is a newer concept first defined by Hwang (1995) and considered analogous to the definition of self-esteem (self-regard) as applied to others (Bowles et al., 2013; Busse and Flowers, 2017). Both attitudes are closely related to self-concept theory, described as “an active structure that organizes and gives meaning to past and current experiences, provides goals and standards for behavior, and motivates future choices and actions” (Harter, 2012, cited in Keefer, 2015, p. 10).

In a review of self-report assessments for emotional competencies, Keefer (2015, p. 9) comments that “self-concept theory is conspicuously missing from the bulk of empirical research. I see this as a major oversight ….” This assertion is supported by applied research on self-efficacy (Bandura, 1997), growth mindset (Dweck and Leggett, 1988), core self-evaluation (Judge and Bono, 2001), and other self-concept theories discussed later in this paper, that self-belief can greatly influence how successfully an individual may harness their potential to achieve effective outcomes and performance. Despite this view, there has been widespread criticism of measures that include self-concept scales such as self-regard as “irrelevant variables” that are “unmooring the concept” of EI (Mayer et al., 2008, pp. 504 and 508). Far from being irrelevant, it is argued that the attitudes of self-regard and regard for others can provide strong foundations for the intrapersonal and interpersonal streams of EI/EE that may positively influence behavior. Other established instruments such as the Bar-On EQi (1997), the EQi 2.0 (Multi-Health Systems, 2011), and the TEIQue (Petrides et al., 2007) include the concept of self-esteem (self-regard), but do not include regard for others. This may be considered a significant omission, as the degree to which we value and accept others may greatly influence our social intentions, interaction, and behavior toward others. On this basis, it is proposed that the UPR attitudes of self-regard and regard for others may form underpinning attitudes to the intrapersonal and interpersonal streams of EI/EE.

Both attitudes may be incorporated within the dual-processing model shown in Figure 1 as either implicit or explicit attitudes (Greenwald et al., 2002). An individual with higher implicit regard for others (IA) may be less judgmental of others, allowing them to better perceive and understand the emotions of others (EI^a^), and in turn, be more open and empathic in their behavior (EE^h^). Or an individual with lower implicit self-regard (IA), may repress uncomfortable feelings (EI^a^) and become more rigid and defensive in their behavior (EE^h^). There may also be a reciprocal influence where changes in behavior influence UPR attitudes. For example, prosocial behavior (EE) has been shown to evoke positive emotions (EI^c^; Aknin et al., 2012) and enhance well-being (EA; Tabassum et al., 2016). Furthermore, there may be interaction between the self and other dimensions; for instance, it may be easier to empathize with the feelings of others (Interpersonal) if one has experienced and become aware of similar feelings in oneself (Intrapersonal; Gallup and Platek, 2002). Appling intrapersonal and interpersonal UPR attitudes to the dual-processing model may increase its complexity, but potentially provides a more complete and accurate representation of EI/EE in practice.

### 3.3. An ethical basis for EI/EE

Another area where the UPR attitudes may be of relevance is in the ethical application of EI/EE. Ethical considerations have been of ongoing concern within the EI literature, particularly for employment, leadership, and organizational settings (Schlegelmilch and Thomas, 2011). Well before the popularization of EI, Goffman (1969) described the strategic manipulation and control of emotions to achieve personal gain, and Caruso and Salovey (2004, p. 171) observe too that “A manager who is expert in managing emotions can use the ability to manipulate employees.” Kilduff et al. (2010) describe how the strategic disguise of one’s own emotions and the manipulation of others’ emotions may be used for self-serving purposes such as career ambition, or as they put it “getting ahead involves leaving others behind” (p. 146). Carr (2000, p.31) argues that the value of EI “is dependent on the moral end which it serves” and Segon and Booth (2015) urge an ethical basis for EI, highlighting competency EI models as lacking ethical foundations. After careful examination of the Emotional Competency Inventory (ECI) and ESCI (Sala, 2002; Boyatzis, 2007) scales and framework, they conclude that any ethical outcome is “a matter of moral luck” and certainly not part of the ECI competencies themselves. This, they suggest, leaves managers and leaders open to potential decisions and actions that are unethical, citing several cases of how the “corporate psychopath” (Babiak and Hare, 2006) may display emotional competencies yet engage in corrupt and unethical practices. They further propose that attitudes, a key component of competencies, should be included within EI models, as they “enable the knowledge and behavior (competencies) to be applied in a way that demonstrates genuineness and authenticity” (p. 790). In current models of EI and EE, there is little reason to assume that higher emotional intelligence will necessarily produce more moral and ethical behavior, just as having higher cognitive intelligence does not confer greater moral values upon an individual.

Attitudes correspond closely with the expression of individuals’ values (Ajzen, 2007), and moral values reflect a person’s ethical orientation. The inclusion of attitude as a basis for EI and EE presents an opportunity to instill ethical foundations in such models. One approach to EI/EE that may facilitate ethical behavior is the nine-layered pyramid of EI (Drigas and Papoutsi, 2018, 2021) in which the higher stages of EI development include self-actualization and transcendence, both of which relate closely to the UPR attitudes. According to the authors, “Self-actualizers feel empathy and kinship toward humanity” and “transcendence is strongly correlated with self-esteem, emotional well-being and global empathy” (p. 8). Self-transcendence also forms part of the Schwartz (2012) theory of basic values and is described as “enhancement of others and transcendence of selfish interests” (p. 9). A potential limitation of the pyramid model is that the higher levels of EI development are dependent on successful transition through the earlier stages, such as emotional recognition, perception (ability-EI), empathy, and social skills (behavioral-EE). These may not be easily achieved if an individual already holds hedonistic or self-serving attitudes.

Another approach to EI/EE that has ethical foundations is the Emotional Intelligence Profile (EIP; Maddocks, 2018). It includes a set of eight foundational attitudes or guiding principles that may facilitate more emotionally intelligent and ethical behaviors. The primary principle, “however you and others are, is okay,” reflects Unconditional Positive Regard and is represented by the EIP scale facets of Self Regard and Regard for Others. A second principle, “People have a natural tendency toward growth,” is drawn from the term Physis (Aristotle, Physics, Book II, Chapter 1), that all living things, including people, are endowed with innate resources to thrive and grow. Adopting this philosophical assumption may encourage greater belief in others, and a more positive, supportive, and encouraging interpersonal environment. Another EIP principle, “people are different,” is taken from the phenomenological position that people experience the world differently, which may encourage greater awareness, understanding, and appreciation of others and their individual differences.

The main thrust of this position is that having higher regard for others will promote more ethical EI/EE. Competencies such as awareness of others and empathy may enable an individual to “read and understand the motivations of others” (Bar-On, 2000), but may be utilized for entirely different purposes depending on whether the individual’s intentional attitude is self-serving (low regard for others) or altruistic (high regard for others). It would seem incompatible for unethical EE behaviors to be present in an individual who has unconditional regard for others. Prosocial attitudes are capable of guiding prosocial actions across different domains (e.g., Balconi and Canavesio, 2013). For example, studies of empathy, prosocial, and moral identity (i.e., a person who believes that helping others is at the core of who they are), have found these attitudes to strengthen readiness to engage in prosocial and ethical behavior (Hardy and Carlo, 2011; Davis, 2015; Hertz and Krettenauer, 2016). Self-regard too may impact moral behavior. Longitudinal research over 30 years (summarized in Kaplan, 1995) shows direct and indirect effects of low self-esteem (also described as negative self-attitudes and self-derogation) on deviant behavior. The proposition that UPR attitudes support ethical EI practices is consistent with the position held by Segon and Booth (2015, p. 790) that “ethical management” should be central to the measurement of EI and that ethical attitudes help demonstrate ethical behaviors.

### 3.4. Balance between the extremes of EI/EE

Another related concern that may be addressed by the UPR attitudes of self-regard and regard for others is non-linearity or the “dark side” of EI/EE constructs. EI is generally considered to fall under the umbrella of positive psychology (Salovey et al., 2002) and there is indeed substantial evidence for its positive impact (Furnham and Petrides, 2003; Austin et al., 2005; Day et al., 2005). However, Kilduff et al. (2010 p. 147) express concern at the “overly-positive celebration of EI” and the imbalance of research focusing almost exclusively on the prosocial aspects of EI (Antonakis and Dietz, 2010). Dasborough et al. (2021) call on scholars for greater balance, to examine negative as well as positive outcomes of EI in future research endeavors. Following their meta-analysis on EI and the Dark Triad, Miao et al. (2019) recommend that “future studies may investigate whether there is a ‘too much of a good thing’ effect of EI” and “explore the possibilities of curvilinear relationships” (pp. 195–6). This concurs with an earlier review of the literature, which “points to the possibility of ‘optimal’ levels of EI – both within and across constructs” (Davis and Nichols, 2016, p. 1). Several commentators have argued the benefits of exploring non-linear aspects of personality and behavior (Benson and Campbell, 2007; Le et al., 2011), which remains a contentious issue (Walmsley et al., 2018), and has rarely been applied to the measurement of EI or EE (Maddocks et al., 2020).

The potential for non-linear patterns of EI/EE, which recognize both optimal and sub-optimal polarities, may in part be represented by the UPR attitudes of self-regard and regard for others. Overuse, or ‘too much’ of an EI/EE facet may be driven by a combination of high self-regard and low regard for others, suggesting that an individual has a degree of arrogance (i.e., I am more valuable than others).^2^ Examples of this may include being too assertive (aggressive), too independent, mistrusting, emotionally over-controlled, and overly optimistic. The bipolar opposite to this, ‘too little’ EI/EE, may be driven by a combination of low self-regard and high regard for others (i.e., others are more valuable than me) and be associated with self-deprecating tendencies such as passive, dependent, over-trusting, emotionally under-controlled, and pessimistic. The optimal or balanced position that represents higher EI/EE would be reflected in the combination of high self-regard and high regard for others (i.e., holding the UPR attitude of value and acceptance toward oneself and others). Examples of this may include being assertive, emotionally balanced, cautiously trusting, and realistically optimistic. Attaining an optimal level of EI/EE is consistent with Aristotle’s human virtue of finding “the mean between the extremes” (De George, 1999) in which there is a “golden mean” that provides a form of self-control and moderation between the extremes and excesses that may cause harm and disadvantage to individuals and others in society. Achieving a virtuous balance between extremes also aligns with the premise that UPR attitudes support an ethical basis for EI/EE.

By representing both the optimal and sub-optimal polarities of EI/EE, the UPR attitudes may also reflect some of the behavioral variation inherent in emotion-related facets of EI and EE. Several scholars express concern that the use of psychometric questionnaires to measure subjective emotions and EI is too restrictive (Ashton-James, 2003; Lindebaum, 2009) and argue that “boxing” emotions by numbers bears crude resemblance to the complexities of a person’s affective life (Fineman, 2004). This concern may partly be considered through the lens of UPR attitudes. For example, a common facet of behavioral-EE is conflict handling, which in theory will manifest as either optimal (assertive) or sub-optimal (passive or aggressive) behavior. However, in practice, there are often more subtle, dynamic, and variable interactions between the three elements of conflict handling. For instance, an individual who is passive may also become aggressive, due in part to “surface acting” i.e., the emotional labor of withholding feelings (Hochschild, 1983). This rebound from one extreme to the other may also occur in several other facets of EI/EE such as trust – an individual who is over-trusting is more likely to be let down by others causing them to become mistrusting, and optimism – an individual who is overoptimistic is more likely to experience failure which might cause them to feel despondent and pessimistic. Given that the UPR attitudes can capture the optimal and sub-optimal elements of EI/EE, they may be incorporated within models and measures of EI/EE to reflect the dynamic variability in these facets.

As discussed, the UPR attitudes of self-regard and regard provide a coherent foundation to the intrapersonal and interpersonal pillars of EI/EE that offers several constructive benefits to understanding and applying EI/EE. It is not suggested that all aspects of EI/EE be drawn back to the UPR attitudes, however, given the emphasis on EI/EE being an adaptive quality, it would seem incumbent on theorists in this field to represent the ethical, optimal, and sub-optimal elements of EI/EE within a coherent and integrated model.

### 3.5. Supporting the development of EI/EE

A further benefit that an attitude-based approach to EI and EE offers is that attitudes are malleable and can be developed (Petty and Cacioppo, 1986; Chaiken, 1987), more so than may be attributable to the intelligence components of EI (Mayer and Cobb, 2000) or the more “static nature” of trait EE facets (Côté, 2014; Alessandri et al., 2015, p. 27). A trigger for the early popularization of EI was the publication, “Emotional Intelligence; Why it can matter more than IQ” (Goleman, 1995), which captured the interest of the business world. This was seen as an egalitarian rebuttal to “The Bell Curve” of Herrnstein and Murray (1994), which argued the importance of IQ for understanding social class in society. IQ was seen by many as hard, elitist, and difficult to develop, while EI (or EQ) was seen to be kind, and something that all people could develop. Although many of the grander claims by Goleman have since been moderated (Emmerling and Goleman, 2003), there is growing evidence that aspects of EI/EE can be developed. Two meta-analytic studies have demonstrated that EI and EE can be improved through training interventions (Hodzic et al., 2017; Mattingly and Kraiger, 2019). The authors of the second study conclude; “The moderate and positive effect of training on EI supports the malleability of this construct, allowing us to infer that EI is trainable.” (p. 152).

Despite emerging evidence for the trainability of EI and EE, it would be difficult to attribute this to changes in either aspects of intelligence or personality traits. Intelligence is broadly regarded as a stable attribute over time (Kagan, 1980), as are dispositional personality traits (Costa and McCrae, 1997). However, there may be elements within both models of EI and EE that are more open to development, in particular elements of self-concept, attitude, and self-belief (Bandura, 1997; Marsh and Craven, 2006; Forgas et al., 2010), that may reinforce self-perceived emotional competence (Keefer, 2015). A primary example is beliefs in emotional self-efficacy, which have been found to help individuals express positive emotions, regulate negative emotions, promote prosocial behavior, and support their self-esteem (Alessandri et al., 2015). Similarly, core self-evaluation (CSE), which represents the general and fundamental beliefs individuals hold about themselves, such as their self-esteem and self-efficacy (Judge and Bono, 2001), has been shown to have strong links to trait EI (e.g., *r* = 0.78, Kluemper, 2008) and be predictive of positive work outcomes such as job satisfaction, job performance, and well-being (Caprara and Steca, 2005).

A related avenue of self-development for EI and EE are implicit theories (Dweck and Leggett, 1988) which hold that people who have a growth mindset (incremental theories) and believe that emotions, intelligence, and behavior can be changed, are more likely to put in the hard work and strategies to make this happen (Aronson et al., 2002; Blackwell et al., 2007). Implicit theories of emotions have shown that those holding incremental theories more frequently use cognitive reappraisal as an emotional regulation strategy, experience more positive and fewer negative emotions, receive greater social support, are more likely to use mastery-oriented strategies rather than helpless strategies, and harbor higher expectations of success (Tamir et al., 2007; Burnette et al., 2013; De Castella et al., 2013). A growth mindset has also been associated with higher EI. Perreault et al. (2014) found that general self-determination (GSD) could account for individual variations in EI, and other studies suggest that people’s implicit theories about EI may influence their emotional abilities (Cabello and Fernández-Berrocal, 2015).

Another self-development methodology linked to attitude and EI is mindfulness, described as an attitude characterized by nonjudgment of, and openness to, current experience (Bishop et al., 2004; Brown and Ryan, 2004). A systematic review of an eight-week mindfulness-based stress reduction program (MBSR) found that MBSR led to changes in the brain’s amygdala consistent with improved emotional regulation (Gotink et al., 2016). A meta-analytic review (Miao et al., 2018) found EI had a statistically significant association with trait mindfulness which increased with age, suggesting that mindfulness practice encourages the development of key abilities and competencies associated with EI. As an approach to self-development, mindfulness resonates well with the view of Lindebaum (2009) that changing one’s attitude to focus on the present is synonymous with EI and in turn, leads to behavioral change. These examples lend credence to the view that attitude plays an important role in the development of EI and EE.

Further endorsement for attitudes supporting EI/EE development can be drawn from the benefits previously discussed. The case was made for there being a close relationship between attitude, ability-EI, and behavioral-EE, and that changes in attitude may facilitate a corresponding change in EI and EE. Moreover, changes in EI/EE may be more sustainable and enduring when congruent with a person’s attitudes. This is supported by dual processing theories of EI/EE which, as proposed, may be extended to include conscious (explicit) and automatic (implicit) attitudes. A key feature of dual-processing, essential to personal development, is that conscious procedural practices may become skilled, habitual, and enduring, and that explicit attitudes may become implicit and automated through repetition and experience over time (Gawronski and Bodenhausen, 2006). In which case, greater emphasis could be placed on building automaticity to facilitate longer-term, sustainable change in behavior, much sought after by organizations investing in employee development. A limitation of implicit attitudes is that they are unconscious and not open to self-report, which may render them difficult to change. However, being unconscious and automated does not mean that implicit attitudes and processes are inaccessible and cannot be brought into conscious awareness or developed. Through introspection and self-observation, a person may become aware of their feeling, thinking, and behavioral patterns, giving clues as to their unconscious attitudes (Bem, 1972, p. 2) which may in itself cause them to change their attitudes (Wilson et al., 1989). Self-awareness training may also help an individual become aware of other unconscious ‘blind spots’ such as automatic emotional triggers, defensive habits, and unconscious biases, that may further support personal development. When considering what attitudes facilitate EI/EE, the case was made for a humanistic approach that encourages ethical behavior. Humanistic attitudes are inherently developmental, as they start from the assumption that people are predisposed toward growth (Maslow, 1962; Alicke and Sedikides, 2009). This was identified in the UPR attitudes of self-regard and regard for others, the combination of which help differentiate between optimal and sub-optimal aspects of EI/EE. Greater understanding and awareness of the antecedents to maladaptive behavior may enable an individual to address the causes of lower EI/EE and facilitate their personal development.

Given that attitudes play an important role in driving emotions, fueling cognition, and influencing behavior, they are noticeably absent from models of EI and EE. This may be seen as a significant gap for a concept that has considerable potential for enhancing self-awareness and personal development. As proposed, attitudes may be incorporated as foundational elements to EI/EE that promote personal and interpersonal improvement.

## 4. Conclusion

In this paper, it has been proposed that attitudes provide an important foundation for integrated models of ability-EI and EE traits and competencies. These models vary in their proposition, some present EI and EE as parallel processes, and others as sequential stages. One reason for this may be a lack of differentiation between the ontological status of EE facets, some of which may be antecedent to EI (such as attitudes, beliefs, and values), and others’ outcomes of EI (such as behavioral skills and habits). Given the close relationship between attitude, emotion, cognition, and behavior, it is argued that attitudes play an important role as potential precursors of EI and EE which should be incorporated within integrated models and explored through further research.

Several benefits may be derived from the inclusion of attitudes as a basis for EI/EE that help address some current concerns with existing approaches. The first of these is a dual-processing model for EI/EE, an aspect often overlooked by researchers in this field. It was proposed that explicit attitudes may underpin the conscious processing of EI/EE, and implicit attitudes may underpin the automated processing of EI/EE. The combination of these three components (attitude, EI, and EE) and their two streams (conscious and automated) builds upon existing integrated models of EI/EE. The relationship between attitude, EI, and EE has still to be tested empirically, for which this paper may provide a stimulus for initial hypotheses.

Another concern that may be addressed by underlying attitudes, is providing an ethical basis for EI/EE. Drawn from humanistic psychology and the principle of Unconditional Positive Regard (UPR), two core attitudes were recommended, self-regard and regard for others. These attitudes may also underpin the two core pillars of EI/EE, intrapersonal (self) and interpersonal (others), a distinction often missing from other integrated approaches to EI/EE. The combination of both attitudes may address other concerns within the EI/EE literature, namely, they may differentiate the optimal and sub-optimal elements of EI/EE, provide balance between the extremes of too much and too little EI/EE, and represent dynamic variability inherent in some facets of EI/EE.

A broader implication and further benefit of an attitude-based approach to EI/EE is that it may better support the application of EI and EE for personal development. Specifically: attitudes may be more malleable than the intelligence and trait components of EI/EE; individual changes in EI and EE may be more sustainable when they are congruent with underlying attitudes; attitudes may help to understand and address the antecedents of maladaptive EI/EE; and changes in EI/EE are more likely to become automated and habitual if they are embedded as implicit attitudes. Providing attitude-based coaching and development in EI/EE could be a valuable avenue for further exploration and experimental research.

## Data availability statement

The original contributions presented in the study are included in the article/supplementary material, further inquiries can be directed to the corresponding author.

## Author contributions

The author confirms being the sole contributor of this work and has approved it for publication.

## Funding

This study was funded by Talogy. The funder was not involved in the study design, collection, analysis, interpretation of data, the writing of this article, or the decision to submit it for publication.

## Conflict of interest

JM was employed by company Talogy.

## Publisher’s note

All claims expressed in this article are solely those of the authors and do not necessarily represent those of their affiliated organizations, or those of the publisher, the editors and the reviewers. Any product that may be evaluated in this article, or claim that may be made by its manufacturer, is not guaranteed or endorsed by the publisher.

## Footnotes

^1^Attitudes are closely intertwined with other social-cognitive constructs that sit within the lower-order levels of personality space (McCrae and Costa, 2008), such as beliefs and values. According to Schwartz (2012) a crucial difference is in how they vary: attitudes are evaluative and vary on a positive/negative scale, beliefs vary in how certain we are that something is true, and values vary in importance as guiding principles in life. All three share a common theme of influencing decisions and motivating behavior.

^2^Although these behaviors present as having high or excessive explicit self-regard they are also potentially masking low implicit self-regard (Bosson et al., 2008), i.e., being critical of others may be an unconscious coping strategy to avoid being critical of oneself. This also helps explain the apparent contradiction of having "too much of a good thing."
